# Changing the narrative: A qualitative study on the impact of media portrayals on people with schizophrenia

**DOI:** 10.1371/journal.pone.0335008

**Published:** 2025-10-31

**Authors:** Celia Martí-García, Casta Quemada-González, Carlos Aguilera-Serrano, Yolanda Mejías Martín, M. Paz García-Caro

**Affiliations:** 1 Department of Nursing and Podiatry, Facultad de Ciencias de la Salud, Universidad de Málaga, Málaga, Spain; 2 Department of Mental Health, University General Hospital of Málaga, Málaga, Spain; 3 Instituto de Investigación Biomédica de Málaga (IBIMA), Campanillas, Málaga. Spain; 4 Hospital Universitario Virgen de las Nieves, Granada, Spain; 5 Biosanitary Research Institute of Granada (IBS. Granada), Granada, Spain; 6 Department of Nursing, Universidad de Granada, Granada, Spain; 7 CIMCYC, Mind, Brain and Behavior Research Center, Campus de Cartuja, University of Granada, Granada, Spain; University of Missouri School of Medicine, UNITED STATES OF AMERICA

## Abstract

The stigma surrounding schizophrenia, heavily shaped by media portrayals, significantly affects individuals’ lives, often delaying help-seeking and treatment. These negative representations can lead to social rejection, isolation, and worsened mental health outcomes. This qualitative study delves into the lived experiences of individuals diagnosed with schizophrenia, exploring how public perceptions of mental disorders, particularly schizophrenia, influence their health journeys and daily lives. Rooted in Gadamer’s hermeneutic phenomenology, the study involved in-depth interviews with 10 participants diagnosed with schizophrenia. Data were analyzed using ATLAS.ti software to uncover key themes in their experiences. Four themes emerged. Media Portrayal highlighted how negative depictions of schizophrenia, such as violence, reinforce societal stigma. This leads to Self-Stigma, where people diagnosed with schizophrenia internalize these views, hindering help-seeking. Social Support emphasized the importance of family and friends, although stigma sometimes complicates these relationships. Healthcare Provider Relationships pointed to frustration with frequent staff changes, undermining trust in care. Participants suggested Professional Training in empathy and a Shift in Media Representation to improve perceptions of schizophrenia and Mental Health Literacy. Media representations and societal stigma significantly impact the health-illness process for individuals with schizophrenia. More accurate portrayals in the media, combined with greater awareness and empathy from healthcare providers, could reduce stigma and improve treatment engagement and trust.

## Introduction

The study of stigma related to mental disorders is not a new phenomenon; however, current data do not seem to lead to a deeper understanding of the underlying meanings that perpetuate a problem negatively impacting the health of individuals living with mental disorders and their families [1]. Among various mental health conditions, schizophrenia is particularly susceptible to stigma, with diagnostic labels significantly influencing rejection attitudes [2].

The different ways in which people with mental disorders experience discrimination may affect them in diverse forms [1]. Including other important, yet less explored, manifestations of stigma could yield new insights and perspectives on the problem. In this context, cultural stigma plays a relevant role as individuals with schizophrenia often possess a stigmatized identity that may restrict their opportunities [3]. Culture can help us understand how stigma affects these individuals by exploring specific issues that may have predictive value for the social functioning of people diagnoses with schizophrenia [4]. The public image of people with mental disorders, particularly those with schizophrenia, is a culturally specific issue that, although previously explored, still requires further investigation to fully understand its implications and effects.

## Background

Mental disorders are a reality that, according to the WHO, affects approximately 970 million people worldwide, with about 24 million individuals affected by schizophrenia. Most disorders originate in early ages, and failing to treat them in a timely manner leads, among other things, to a reduced life expectancy, resulting in significant economic costs. Additionally, there are social costs, including lower quality of life, family expenses, and, of course, stigma and discrimination [5].

Despite available treatments, nearly two-thirds of those affected never seek professional help. This seems to be related to various factors, such as the desire to manage the situation independently, a low perception of the need for help, limited health literacy, economic factors, cultural values (including specific frameworks that attribute the origin of psychosis to spiritual or religious causes), previous negative experiences within the healthcare system and the stigma these people diagnosed with schizophrenia endure from society [6–8].

For some individuals diagnosed with schizophrenia, mental illness may be perceived as a personal failure, contributing to the development of what is known as self-stigma. The internalization of negative stereotypes can lead to stress, increased susceptibility to depression, reduced self-esteem, and the worsening of symptoms. It may also undermine adherence to treatment and its continuity [9]. Stigma can trigger a range of responses from society, as well as from individuals living with the condition, their families, and the healthcare personnel who support them. These responses can have repercussions for the person diagnosed with schizophrenia and their family, including physical and mental health issues, social exclusion, and additional barriers to recovery [10].

Mental illness was often depicted as divine punishment, demonic possession, or a cause for exclusion, a view that may have been exacerbated in recent years by the stereotypes perpetuated by the media. Film, press, and television present a manipulated image designed to capture public attention, employing the sensationalism typical of entertainment programs [11]; this portrayal often emphasizes rarity, social incompetence, and a propensity for violence [12]. It is important to note that, for most of the population, this biased image is the only representation they have of what a mental disorder is, accepting from a very early age that the villain in movies is often portrayed as a mentally ill person. Even among healthcare professionals in mental health, where one would assume a certain level of training, stigmatizing attitudes persist regarding this skewed image of mental disorders, particularly towards schizophrenia [13].

Previous studies on the impact of the public image of mental disorders have established the influence of this image on the population’s perceptions of mental disorders [14], recognizing the person with a mental disorder as an unpredictable, incompetent, and dangerous individual. This perception can be modulated, for example, through contact with individuals with lived experience [15] or even through the use of films [3]. This socio-cultural construct affects the individual diagnosed with a mental disorder, who internalizes these characteristics as part of their self-concept, impacting their self-esteem, perception of competence, and expectations about treatment.

Despite the impact that the public image of mental disorders can have on individuals diagnosed with these conditions, their families, and the professionals who care for them, previous studies analyzing stigmatizing images have primarily focused on describing the frequency with which films, television programs, or the press depict mentally ill individuals in a derogatory and biased manner. Those that have attempted to delve deeper into how individuals living with mental disorders perceive the societal image of mental illnesses, while emphasizing that media representation is a significant source of distress for those affected, do not seem to provide an explanation beyond categorizing it as a negative and stigmatizing image [16]. On the other hand, professionals who care for these individuals identify the public image of mental illnesses as one of the most stigmatizing experiences for those suffering from schizophrenia, equating it with the stigma faced by those directly affected in the context of direct social interaction [17].

Previous research has identified existing gaps in the literature, particularly the limited number of qualitative studies exploring how individuals with mental disorders perceive and experience stigma in their daily lives [18]. Gaining insight into these experiences is essential for developing effective strategies to address this issue [1]. Despite increasing awareness of the relevance of stigma, the voices of those directly affected have often been overlooked in theoretical frameworks and empirical research. In particular, media portrayals of mental illness remain underexplored, even though they play a key role in shaping public perceptions. There is a pressing need for in-depth investigations into how these representations influence the mental health and recovery process of those affected, with the ultimate aim of reducing stigma [19]. For this reason, there is a need to more specifically understand the impact of the public image of mental disorders on those navigating mental illness in their everyday lives, and to potentially identify specific details about this representation that could serve as a foundation for future studies on the conceptualization of stigma and possible measures to combat it. In this regard, studies utilizing qualitative methodology are particularly well-suited, as they help deepen the understanding of subjective experiences, placing the person’s lived experience at the center of attention.

Therefore, the objective of this qualitative study is to explore and understand how individuals diagnosed with schizophrenia perceive the impact that the public image of mental disorders has on their health-illness process and personal lives.

The research question posed is as follows: “How do individuals with schizophrenia perceive and experience the impact of the public image of mental disorders on their health-illness process and personal lives, in the context of current society and the media representation of schizophrenia?”

## Materials and methods

The design of this study was based on a qualitative approach grounded in Gadamer’s hermeneutic phenomenology [20], which posits that understanding the meaning individuals assign to their experiences is essential. This methodological framework was particularly suited to our research question, as it allows for an in-depth interpretation of how individuals diagnosed with schizophrenia make sense of their lived experiences. By emphasizing the role of dialogue and the fusion of horizons between participants and researchers, Gadamerian hermeneutic phenomenology offers a coherent epistemological foundation for exploring the impact of sociocultural representations on the health–illness process. This approach involved an interpretive process mediated by our pre-understanding, culture, tradition, and history [20]. To carry out the study, the phases proposed by Fleming et al. [21] were followed, which included formulating a research question, identifying the pre-understanding of the problem, understanding through dialogue with participants, interpreting the text, and establishing reliability.

In the first phase, the research team determined the relevance of the formulated question and its alignment with the selected methodology. Regarding the researchers’ prior understanding of the study topic, the team consisted of five researchers (one male and four females) with clinical experience and expertise in qualitative research methodology. The five researchers conducting the study were all trained nurses, three of whom were also specialists in mental health. Four held PhDs, and their academic backgrounds also included degrees in anthropology (three researchers) and psychology (two researchers), contributing to a multidisciplinary perspective. Two of the researchers worked exclusively in academic settings, while the other three were primarily involved in clinical practice—two of whom also held academic. These researchers played a crucial role in data analysis and the drafting of results, ensuring that the study reflected a deep and accurate interpretation of the participants’ experiences. The recommendations of the COREQ guidelines were followed [22] to develop the manuscript.

### Participants and context

Participants were recruited through purposive sampling. The inclusion criteria for the study were that participants must be individuals diagnosed with schizophrenia, aged 16 years or older, who had no active symptoms at the time of the study, and who did not exhibit cognitive impairments or language limitations that could influence their participation. Additionally, they needed to provide informed consent to take part in the study.

On the other hand, individuals were excluded from the study if they had a diagnosed disorder related to substance abuse (alcohol or illegal drugs), if they had an intellectual disability, or if they had discontinued treatment without being formally discharged by a healthcare professional.

Clinical stability was assessed using the Brief Psychiatric Rating Scale (BPRS), requiring a score of ≤2. Participants also needed to have had no hospitalizations in the past six months and no significant changes in their treatment regimen in the previous three months. Cognitive impairment was evaluated using the Pfeiffer questionnaire alongside the patient’s medical history.

To facilitate recruitment, two mental health nurses and members of local associations who had in-depth knowledge of potential participants’ profiles and clinical trajectories collaborated in the process. This approach allowed for the selection of a diverse sample in terms of age, gender, and clinical background, aligned with the study’s aim to ensure maximum heterogeneity when capturing perspectives on the research topic.

This study was conducted in Andalusia, a region in southern Spain where mental health services are primarily delivered through the public healthcare system, the Andalusian Health Service (SAS). The SAS follows a community-based model coordinate by mental health departments that include Community Mental Health Units, hospitalization units, day hospitals, and rehabilitation services, all integrated with primary care to provide universal, publicly funded coverage [23].

Although mental health care is universally accessible via the National Health System (SNS), access to psychological services—particularly therapy—can be hampered by long waiting lists and administrative delays. For instance, only about 27% of individuals seeking help through public services receive care within a month, while another 64% must wait from one to over three months [24].

A total of thirteen individuals who met the inclusion and exclusion criteria were invited to participate in the study, and ultimately ten agreed to take part. Of the three who declined, two cited fear of being identified, while one experienced a worsening of symptoms in the days prior to the schedule interview.

The study guided its sampling process by the principle of data saturation, continuing to recruit participants until the analysis indicated that no new significant codes or categories were emerging from the interviews. This point was reached after the tenth interview, when the research team observed that the last transcripts did not provide new insights or modify the emerging thematic structure. Saturation was assessed iteratively during data analysis, in accordance with hermeneutic principles, where the depth and richness of interpretation took precedence over the number of participants.

### Data collection

Individual in-depth interviews were conducted with people diagnosed with schizophrenia to allow participants to communicate their experiences, feelings, and emotions. The interviews were conducted from January to July 2024 and lasted an average of approximately 45 minutes.

Participants were recruited through the Mental Health Unit of the Regional Hospital of Málaga, as well as through the associations AFESOL and AFENES. If individuals expressed interest in participating, they were provided with an information sheet and informed consent form. Upon acceptance, they were contacted by phone to explain the procedure and to schedule the date and location of the interview according to their preferences. All interviews were conducted in private rooms located either within hospital or primary care facilities, or in the local mental health associations that participants regularly attended. The suitability of these locations for data collection was reviewed and approved in advance by the relevant ethics committee.

The interviews were conducted by two researchers with training and experience in qualitative research. In addition to the interview data, other significant information was collected. Relevant sociodemographic and clinical data were also gathered.

As part of the second phase of Fleming et al. [21], the researchers engaged in a reflexive process to identify their pre-understandings of the phenomenon, drawing on their clinical and academic experience in mental health care and research. This reflection was enriched through a targeted review of existing literature, which served not to define analytical categories, but to guide the development of the interview guide and to deepen the initial understanding of the phenomenon in line with Gadamerian philosophy. The resulting interview guide, composed of open-ended questions, was designed to encourage detailed responses from participants (see Table 1). All interviews were audio-recorded with the participants’ consent, and field notes were taken to document non-verbal cues and contextual information relevant for the interpretative analysis.

**Table 1:** Interview guide.

Stage	Subject	Content/example Questions
**Introduction**	Motives/Purpose	Understanding the impact of the public image of mental disorders on individuals’ health processes for providing support to others and proposing potential modifications to the media representation of mental health issues.
	Ethical issues	Inform about volunteering, recording, consent, withdrawing.
**Opening**	Introductory question	Film, press, and television reflect a public image of mental disorders and the individuals who suffer from them. In your opinion, what image do you think society has of people with schizophrenia?
**Development**	Conversation guide	What impact do you believe this image has had on your life and health process so far? What role did the public image of mental disorders play in your decision to seek help? How do you think the public image of mental disorders has affected the healthcare personnel who care for you, as well as your family and friends? What impact do you think the portrayal of mental disorders has had on your beliefs about yourself, your self-esteem, and how you cope with your illness? What types of images do you think produce the most stigma reactions from your perspective? What types of strategies do you think could be proposed to modify this public image?
**Closing**	Final question	Is there anything else you would like to share with us?
	Appreciation	Express gratitude for their participation. Inform them about how their account will be utilized and let them know they can reach out to us if they have any questions.

### Data analysis

All interviews were transcribed and incorporated into the Hermeneutic Unit for subsequent analysis using ATLAS.Ti 23.0. The software was used to organize the transcripts, support inductive coding, and facilitate the development of themes by grouping codes and retrieving relevant segments.

Before starting the formal analysis, researchers conducted an open reading of all transcripts, noting spontaneous reflections and preliminary impressions in analytical memos. This process allowed them to deepen their understanding of the data and engage in an early dialogue with participants’ narratives.

The analysis followed the phases proposed by Fleming et al. [21]. The third phase involved understanding the phenomenon through dialogue with the participants. During the data collection and transcription process, researchers gained immediate insights into what participants expressed.

These initial impressions were documented using memos, which were then revisited during the subsequent analytical steps. Researchers who did not conduct the interviews listened to the recordings to ensure familiarity with the participants’ voices and enhance reflexivity in the analytical process.

The fourth phase focused on the conversation between the researchers and participants through the text, which involved coding the most significant phrases and assigning codes that were grouped into units of meaning, which were then organized into subthemes and themes.

Throughout this phase, researchers maintained a reflexive attitude, using ATLAS.Ti’s comment and memo functions to document their evolving interpretations and to support the hermeneutic circle between parts and whole. All themes and subthemes were discussed until consensus was reached.

### Rigor

The fifth step of the procedure outlined by Fleming et al. [21] focuses on rigor. To ensure quality in qualitative research, reliability was enhanced by maintaining transparency throughout the research process, documenting a detailed audit trail, and remaining faithful to the participants’ narratives and the context of their experiences. This included the use of triangulation, peer debriefing, and clearly documenting each phase of data collection and analysis.

Credibility was established by providing all participants with the opportunity to confirm the transcriptions and results based on their responses. After drafting the initial version of the findings, participants were contacted by phone. Seven individuals were successfully reached, and among them, three agreed to meet and review the material to confirm the accuracy of the data and interpretations. Additionally, credibility was enhanced through triangulation among researchers.

Two researchers independently coded the data using ATLAS.ti. Initial discrepancies were addressed through reflexive dialogue with a third investigator, who contributed to reaching consensus on conflicting codes and interpretations. When necessary, coding disagreements were resolved by returning to the original transcripts and engaging in interpretive reflection to ensure the most accurate understanding of the participants’ meanings. Once agreement was reached, the full research team held additional meetings to review and refine the final units of meaning, subthemes, and themes, ensuring consistency with the hermeneutic framework. Reflexivity also played a key role, with the research team meeting to discuss the design, data collection, and analysis processes. They listened to the recordings of the first two interviews to refine and improve subsequent interviews.

### Ethical aspects

The protocol was approved by the Biomedical Research Ethics Committee of Andalusia (code ESQUIGMA 1563-N-23). Before participating in the study, each participant received both written and oral information about the project’s objectives and methodology. Participation was voluntary, and individuals were informed that choosing not to participate would not result in any negative consequences. The capacity to provide consent was ensured through the inclusion criteria related to clinical and cognitive stability. All procedures performed in this study involving human participants, including the consent procedure was approved by the Ethics Committee.

While qualitative studies do not pose physical risks, the research team took care to avoid unnecessary inconveniences for participants by adapting data collection to meet their needs and preferences. The study adhered to good clinical practice standards and followed the ethical principles established for human research in the Declaration of Helsinki and its subsequent revisions.

To protect participant confidentiality, names were changed in the interview transcriptions, using pseudonyms or codes (e.g., EP-1, EP-2… EPn), and any identifying characteristics that are not pertinent to the study were modified. Data collected from the study was securely stored on computers accessible only to the research team.

All records complied with current legislation on the protection of personal data.

## Results

A total of 10 participants were included in the study. The demographic details are as follows: 6 participants were male and 4 were female, with an average age of 42.4 (±10.20) years and an average age at the time of diagnosis of 28.9 (±10.82) years. Additionally, 6 participants had previous hospitalizations. Regarding civil status, 70% of the participants were single, 20% were divorced, and 10% were in a relationship. In terms of employment status, 4 participants were actively employed, 3 were unemployed, and 3 had a permanent disability. The educational levels of the participants varied, with some having completed middle or higher education and others having only elementary education. The sociodemographic characteristics of the participants can be seen in Table 2.

**Table 2:** Socio-demographic data of the participants (N = 10).

**Gender**	**Age**	**Marital Status**	**Employment Status**	**Age At Diagnosis**	**Educational Level**	**Place of Residence**	**Last Hospitalization**
F	61	Divorced	Active	51	Middle or higher	Urban	No admissions
F	42	Single	Unemployed	26	Middle or higher	Urban	2012
M	40	Single	Disability	20	Middle or higher	Urban	2019
F	25	Single	Unemployed	19	Middle or higher	Urban	No admissions
M	43	Single	Disability	26	Elementary	Urban	No admissions
F	50	Divorced	Disability	37	Middle or higher	Urban	2011
M	37	Single	Active	34	Middle or higher	Urban	2020
M	31	Married/Partner	Active	19	Middle or higher	Urban	2013
M	50	Single	Unemployed	38	Elementary	Urban	No admissions
M	45	Single	Active	19	Elementary	Urban	2003

To protect participant anonymity, the order of individuals listed in the table does not correspond to the order in which their quotations appear in the manuscript.

Four main themes were identified: (i) The mask of schizophrenia: Media’s distorted lens, (ii) the burden of misrepresentation: the harmful impact of public image on life and health, (iii) Surviving mental health struggles, and (iv) Shifting the narrative: understanding and reversing the stigma of mental illness. Along with their subthemes and units of meaning (see Table 3), these findings provide insights into how individuals diagnosed with schizophrenia experience societal stigma, cope with their condition, and navigate the challenges posed by media portrayals and gender-based differences.

**Table 3:** Themes, subthemes and units of meaning.

Theme	Subtheme	Units of meaning
The mask of schizophrenia: Media’s distorted lens	Stigma in the shadows: fear, rejection, and misunderstanding of schizophrenia	Knowledge, stereotypical beliefs, perceptions, and taboos about schizophrenia; prejudices and rejection towards the illness
	Behind the mask: the narrow and sensationalized view of mental health	Lack of diversity and sensitivity in media portrayals; biased portrayals and untrained informants; inaccessible or technical language; superficial and oversimplified coverage of mental health issues.
	The villain in white: the dark side of treatment	Prejudices and rejection of treatment, and mental health professionals.
	Layers of stigma: the shifting perception of mental illness	Impact of external factors, such as life stage/age on illness perception; differences in stigma based on diagnosis or gender; social rejection influenced by symptom severity and social status.
The burden of misrepresentation: The harmful impact of public image on life and health	When the diagnosis builds walls	The role of social support from friends and family in managing the illness; how media representations affect the care received, self-concept, and interactions with others; the impact of diagnosis on relationships; and the emotional effects of public perception, including frustration, indifference, and feelings of being judged.
	Unspoken struggles: the hidden costs of mental illness stigma	Social isolation, personal and family history factors, the broader impact of illness on family well-being, disruptions caused by acute episodes, challenges in professional and academic life, abuse experiences, and difficulties in securing adequate housing.
	Navigating the system: access and trust in mental health care	Public and private healthcare services, perceived effectiveness of therapies/treatments, hospitalization experience, medication control, need for coercive measures, perception of care quality, professional relationships and attitudes.
Surviving mental health struggles	Shadows of the self	Self-stigma, low self-esteem, emotional distress (confusion, despair, fear, shame), denial of diagnosis, guilt, and illness concealment.
	Resilience in the face of adversity: embracing self-acceptance and support	Support networks, self-acceptance, empowerment, autonomy, resilience, routine maintenance, and therapeutic pets.
Shifting the narrative: Understanding and reversing the stigma of mental illness	Rebuilding the public image of mental health	Enhancing media presence, using influencers, promoting real-life interactions and fostering societal awareness.
	Transforming communication: advocating for empathy and accuracy in mental health	Raising awareness of resources, improving communication skills in professionals, boosting mental health literacy and advocating for a more empathetic and positive portrayal of schizophrenia.

### The mask of schizophrenia: Media’s distorted lens

This theme details how media portrayals shape public perceptions of schizophrenia, reinforcing negative stereotypes and misunderstandings. These distorted portrayals, coupled with a lack of mental health literacy, not only influences how the public views those with schizophrenia but also impacts individuals’ understanding of the illness and their willingness to seek help. Certain external factors are also identified as influencing the perception of schizophrenia, thereby influencing the responses and potential rejection faced by individuals with the condition.

#### Stigma in the shadows: Fear, rejection, and misunderstanding of schizophrenia.

This subtheme focuses on the stigma and misunderstanding that leads to marginalization and a lack of support.

Participants emphasized how deeply entrenched societal attitudes perpetuate fear and misinformation about schizophrenia, resulting in social exclusion and insufficient support networks.

They highlighted the pervasive nature of stigma, often driven by societal misconceptions and a lack of understanding. The public tends to associate schizophrenia with stereotypes, where the illness is linked to extreme behaviors, drugs or criminal actions.

*“Imagine... well, I don’t know... they also associate mental disorders a lot with drugs, right? Like... I don’t see it that way really... Well, yes, it may be that you use drugs and have a mental health issue. But you can use drugs and not have a problem, or you can have a problem and not use drugs. So, associating those things... I don’t see it that way. It’s a bit like generalizing...”* (P1)

This lack of mental health literacy often leads to fear and rejection. People with schizophrenia are frequently perceived as dangerous or incapable of leading a normal life, which only reinforces the stigma.

*“The public image, mostly, is that they are aggressive, that they... if they are not completely capable, they can’t, for example, do certain types of work. That maybe if they are severely affected, they can’t even continue studying or do... what would be considered a normalized life in society.”* (P2)

Furthermore, participants expressed how these misconceptions stem from a general lack of understanding about mental health. This ignorance prevents individuals from receiving the necessary support and treatment, often leading to a sense of invisibility and alienation.

*“Socially, yes, because they are illnesses too. But how they are viewed socially, yes. Because maybe a person who says they take insulin, who injects insulin, that... that’s fine, right? It’s nothing. But, for example, a person who says they... are injected with medication for their mental health process... Well, maybe there, they say... ‘Why do they need to...?’ “* (P8)

These collective misconceptions reinforce negative stereotypes, creating a hostile environment in which individuals with schizophrenia are judged not for who they are, but for the distorted image society has of them. This contributes to a pervasive stigma, shaping how individuals with the condition are perceived and treated by society.

#### Behind the mask: the narrow and sensationalized view of mental health.

Participants highlighted the powerful influence of media portrayals in shaping public opinion about mental health issues, particularly schizophrenia, noting that these representations are frequently biased, oversimplified, and framed for entertainment rather than education. Many expressed that the representation of mental illness in films, television shows, and news broadcasts tends to focus on extreme cases, creating an unrealistic and one-dimensional image.

*“They always show the most striking cases. You can watch a morning show, ‘Espejo Público,’ or any kind of information program, and they always feature people who have had some kind of mishap, a specific moment in their life. So, what is shown on TV never includes people who have recovered, never includes people who are working. What they show is simply that, the juicy content.”* (P1)

Such portrayals contribute to a limited and often alarming view of schizophrenia, reinforcing fear and stereotypes rather than promoting awareness or empathy. Besides, this selective representation fails to show the full range of experiences, especially those of individuals who have recovered or are living normal lives.

*“There are plenty of people who live with schizophrenia and lead regular lives, but those stories never get told”* (P7).

Additionally, participants pointed out that media often use inaccessible or overly technical language that hinders public understanding.

*“...you need to be informed to speak, but no, the information we and the Associations provide doesn’t count. It’s given by politicians. Politicians and others who, although they work in health, aren’t directly engaging with us and the Associations to talk. And moreover, they might speak about things that even I don’t understand, they start saying ‘Mental Health… bla bla bla’, using words that nobody knows.”* (P10)

This disconnection between official communication and lived experience was as widening the gap in mental health literacy. The lack of clarity prevents a deeper understanding of mental health issues and instead perpetuates alienation and stigma.

Finally, the media’s tendency to oversimplify mental health issues means that complex conditions like schizophrenia are rarely explored in depth. Instead, quick fixes or superficial coverage prevail, leaving the public with incomplete and misleading impressions. Participants emphasized that these shallow narratives do not reflect their realities, and instead contribute to fear, ignorance, and marginalization. This superficial approach ultimately perpetuates stigma rather than fostering empathy or understanding.

#### The villain in white: the dark side of treatment.

This subtheme examines the stigma and rejection that individuals with schizophrenia often face towards treatments and mental health professionals. Participants express how societal stereotypes and media portrayals influence their willingness to seek and adhere to treatment. For some, it came with internalized fears and doubts stemming from socially constructed ideas about what it means to take psychiatric medication or be under psychiatric care. These ideas can lead to early struggles with accepting medication, driven by the belief that taking psychiatric drugs equates to being “crazy”.

*“For example, when I was 19 and had my first psychotic episode, I struggled a lot to accept the illness I had and to take the medication because I was convinced that someone who takes medication is... well, someone who is ‘crazy,’ ‘out of their mind.’ I definitely had that stigma”* (P08).

The portrayal of psychiatric treatment in the media also exacerbates these fears and misconceptions. One participant recalls how depictions of mental health care in films, especially those showing psychiatric wards as prison-like environments, made them fearful of receiving treatment.

*“What I saw on TV were psychiatric hospitals that looked like jails. They’d show scenes where they were applying electricity... all negative stuff. I was scared. I thought they were going to lock me up and never let me out”* (P10).

Such depictions, which rarely show recovery or empathetic care, foster fear and mistrust toward treatment options and reinforce the idea that psychiatric care is something to be ashamed of or feared.

In addition to fear, some participants described shame or discomfort in seeking help, not because of the treatment itself, but due to the symbolic weight of what it represents in a stigmatized society. For many, accepting treatment meant acknowledging a socially devalued identity.

This subtheme illustrates how negative portrayals of psychiatric treatment in the media and societal stigma interact to shape individual experiences of care, sometimes delaying or complicating recovery. Participants’ narratives reveal that beyond clinical interventions, addressing these perceptions is key to restoring trust and dignity in mental health care.

#### Layers of stigma: the shifting perception of mental illness.

The stigma surrounding schizophrenia is a multidimensional phenomenon that intersects with various social, cultural and individual factors, which can shape the way individuals are perceived and treated. Participants’ accounts reflect how stigma operates differently depending on how the illness manifests, the identity of the person affected, and the societal lens through which they are viewed. These layered experiences show that stigma is not static but varies according to context and individual characteristics.

Participants highlighted how societal perceptions are influenced by the visibility and severity of symptoms. For instance, individuals with more noticeable symptoms often face harsher judgment and are perceived as “crazier” or more unpredictable, reinforcing a stereotypical and fearful image.

*“I knew him for a while and knew that he had mental health problems. I used to think he was just crazy because I was told he would talk to cars and everything. He would see cars and talk to them... Also, I’ve heard that people with mental health problems tend to move around a lot, well, the crazy ones”* (P09).

In contrast, those who experience less visible symptoms may be perceived more favorably, or remain unnoticed, which in turn offers them a degree of protection from public scrutiny. This reveals an implicit hierarchy of stigma, where those who can “pass” as neurotypical avoid the worst consequences of labeling.

*“It’s not that noticeable, so it doesn’t really show. I have the illness, but it’s very mild. I’ve had it in bouts, they lasted a bit, and then I was fine. I didn’t have that negative sensation, and everyone around me was fine...”* (P06).

Gender was another factor participants identified as influencing how stigma is experience but there were mixed opinions among participants regarding its impact on the perception of mental illness. Some participants believed that women with mental health problems face a double vulnerability, as they are judged not only for their condition but also for their gender. In some cases, past experiences of abuse and social judgment intersected with the stigma of diagnosis, compounding their marginalization.

*“It’s not the same for a woman, still, there are things like this, a woman who’s been abused and doesn’t have mental problems, they see her with more kindness than a woman with mental problems like me, who’s been abused because I’m divorced from my husband, who attacked me with a knife...”* (P10).

On the other hand, some participants argued that men are often portrayed more negatively in the media, particularly by associating them with violence or uncontrolled behavior, thereby reinforcing gendered stereotypes.

*“In the movie ‘Joker,’ for instance… The first time I saw it, I felt bad because you see a man who’s a bit out of his mind, and then, for example, he has a girlfriend, who also loses control a bit, right? So, in that movie, for example, I see that he does crazier things, it tends to show that the man does more crazy things than her. Maybe they both have the same problem, but he’s seen as more out of control and more violent... That’s how I see it “* (P08).

Social status also emerged as a possible protective factor. Being famous or wealthy appeared to mitigate the stigma associated with schizophrenia, allowing some individuals to be accepted despite their diagnosis. This suggests that societal value judgments play a significant role in how the same diagnosis is interpreted.

*“And then, for example, there’s a singer, like Ozzy Osbourne, whom I listen to, I like rock, and he has schizophrenia, and it’s no big deal because he’s Ozzy Osbourne. He’s famous, he’s a musician, so it’s not a problem. Or the guy from Pink Floyd, who also has schizophrenia, right? And nothing happens there, you know?”* (P04).

Together, these accounts highlight the fluid and contextual nature of stigma, showing that how people are treated is not just a result of their diagnosis, but also of how society interprets that diagnosis based on symptoms, gender, class, and their relationship with media narratives. This complex web of perceptions contributes to an uneven landscape of stigma, where certain individuals face greater barriers and social penalties than others. Therefore, stigma is not only based on the illness itself but also on the social context in which it occurs.

### The burden of misrepresentation: The harmful impact of public image on life and health

Media portrayals and societal stereotypes play a significant role in shaping how individuals with mental illness are perceived and treated. These distorted representations not only influence public opinion but also affect individuals’ understanding of their condition, potentially discouraging them from seeking help. The diagnosis of a mental illness can create emotional barriers, with individuals facing frustration, indifference, and feelings of judgment. This stigma can impact relationships with family and friends, as well as interactions with healthcare professionals, complicating both the management of the illness and the experience of receiving care.

#### When the diagnosis builds walls.

Receiving a diagnosis of schizophrenia was described by participants as a pivotal moment that altered how they were seen by others — and how they saw themselves.

While social support was often described as essential to navigating life with schizophrenia, participants emphasized that this support was not guaranteed and could be profoundly affected by the social meaning attached to the diagnosis.

Many participants stressed how important their family and friends are for emotional support and basic daily tasks, underscoring how crucial these relationships are in maintaining stability and quality of life.

*“Right now, I don’t see myself alone, I need the support of my family. I need to be with my mother, walk with her every day, have my father, go out with him in the morning, take the dog out, those little things that seem trivial but sometimes are the ones that matter. It’s those things that stay with you”* (P07).

Another participant emphasized the help they received in difficult moments: *“They have helped me in everything, in all the difficulties I had... for example, I couldn’t drive, they came, or I couldn’t walk, they helped me, ‘I’ll pick you up,’ ‘I’ll help you with whatever you need’”* (P06).

However, the mere disclosure of the diagnosis often marked a turning point in relationships. Several participants shared how once others became aware of their condition, their behavior and attitudes changed, leading to feelings of distance, disappointment, or betrayal.

*“They have a more rooted thought about how they see us. They don’t truly make an effort to know the person who is suffering from schizophrenia. So, they know you in one way, but when they find out you have it, you become different. You’re no longer the same person”* (P02).

*“They don’t treat you the same, you know? They don’t treat you the same anymore, their perspective of you changes. You’re still yourself, but when they know you have problems, they start changing their behavior towards you. I’m not talking about family, I’m talking about friends, acquaintances”* (P07).

These narratives reflect how stigma becomes a barrier not only in public spaces, but also in intimate and everyday relationships. The diagnosis, loaded with social stigma, can overshadow prior bonds, and limit individuals’ ability to maintain a sense of normalcy and connection.

Moreover, participants noted how these changes in others’ behavior contributed to their own internal struggles, reinforcing a sense of being different, misunderstood, or excluded. They pointed out how public perceptions of mental illness and the stigma associated with schizophrenia, also influence their self-concept.

*“But I think it affects more when you are going through a tougher process because... it feels like it influences you more. Like maybe you even see yourself reflected in someone else’s actions, even if you didn’t do what that person did. But just saying ‘someone with schizophrenia’... maybe they don’t even mention the person’s name... they say ‘a man with a mental disorder kills...’ I stay thinking ‘that could be me maybe’ when I’m in a worse situation”* (P02).

Thus, the emotional impact of being diagnosed with schizophrenia can be profound. Many participants expressed feelings of frustration and being questioned by society due to negative portrayals in the media.

*“Sometimes I hear a comment on television, and it’s negative, so it affects you negatively. If you hear a positive comment, it affects you positively”* (P04).

*“I think that… well, it can bother you at any time, really. Even if you’re doing well because you can still say ‘Hey, I’m not that person, okay? But I also have schizophrenia, and I’m not like that... why don’t they show me on the news saying, ‘a person with schizophrenia is doing this,’ and it’s something good”* (P02).

This subtheme reveals that the effects of stigma extend beyond media portrayals or public discourse; they permeate personal spheres, altering the very fabric of social relationships. The “walls” built by the diagnosis are not only psychological but deeply relational—disrupting trust, mutual understanding, and the continuity of support.

#### Unspoken struggles: The hidden costs of mental illness stigma.

This subtheme explores how stigma subtly but powerfully influences various aspects of everyday life for individuals diagnosed with schizophrenia. These struggles often remain invisible, but they reflect the cumulative burden of societal attitudes, internalized stereotypes, and the broader consequences of a distorted public image. The acute episodes or phases of the illness directly affect the person’s self-concept, as one participant shared: *“It influenced my mood, my self-concept... ‘I am good for nothing. I am worth nothing’... Personally, I even dropped out of my studies because I thought, what was I going to do? I couldn’t even think, so why continue?”* (P02). This loss of self-confidence, often linked to the more severe episodes of the illness, can lead to increasing social isolation, as the individual feels unable to maintain their social or work roles.

These personal challenges often extend to the family environment, with many participants acknowledging the strain their illness places on loved ones, emphasizing how stigma affects not only individuals but also their support networks, especially when social misunderstanding and fear of judgment persist. The broader impact on family well-being becomes clear as participants recount the toll that acute episodes take on their relationships.

While some participants highlighted that work was not a major obstacle, others reported limitations in their social relationships due to their illness. One participant mentioned: *“It limits me a bit socially, in relationships with colleagues, but not in terms of work performance”* (P04). Despite this, some noted that their work performance was unaffected, and they were accepted as anyone else, without being labeled for their condition. *“In work, I haven’t had any problems. I perform like a normal person. I’ve been employed without a disability label, and my colleagues value me”* (P01).

Among other issues reported, access to adequate housing after receiving the diagnosis is one of the significant challenges faced by individuals with schizophrenia. One participant described the poor conditions in which they lived: “The place I lived in was not even a proper apartment; it was a tiny house full of cockroaches. In the next street, a guy was stabbed to death, and the following year, another one was killed” (P10). This situation was not attributed merely to economic factors but rather to the way people with schizophrenia are socially devalued, which in turn limits their access to decent housing options.

These accounts highlight how stigma transcends interpersonal interactions and becomes embedded in structural dimensions, shaping access to safety, housing, and overall quality of life.

#### Navigating the system: access and trust in mental health care.

This subtheme explores how stigma intersects with access to mental health services and the trust placed in them. Participants’ accounts reveal that both structural elements (such as continuity of care and access to medication) and interpersonal dynamics (such as professional attitudes and coercive practices) are shaped by—and can reinforce—societal perceptions of schizophrenia.

Participants described their experiences with public and private healthcare services, including both positive and negative aspects. Most participants expressed a sense of relief when receiving care in the public system, emphasizing the valuable support provided by professionals.

*“The first time? I went to a private doctor, but it was very expensive and didn’t help; they just gave me pills”* (P05)

However, some described situations in which they felt dismissed, devalued, or unsafe, pointing out how these experiences were often linked—explicitly or implicitly—to stigma. One of the most commonly reported issues was the lack of continuity in care, marked by frequent changes in the professionals overseeing their treatment: *“I had four psychiatrists in one year. That was something I had difficulty handling”* (P01).

The absence of continuity was experienced not just as an organizational problem, but also as a barrier to trust, leaving some individuals feeling uncertain about the process and treatment, and emotionally exhausted by repeatedly telling their story to new professionals, especially given the stigma already attached to their diagnosis.

Despite these challenges, public healthcare was appreciated for its ability to cover the high costs of medications.

*“The social security covered the medication, which would otherwise be unaffordable for me. I take injections that cost around 500 euros each. I can’t afford that on my own”* (P03).

While some participants reported positive experiences in private healthcare settings -such as shorter wait times- the cost remained a significant barrier. On the other hand, others viewed the public system more favorably due to the broader range of professionals and support services. *“The nurse was a huge surprise; she’s the one who helped me through the whole process”* (P05).

Regarding hospitalization, opinions were mixed. Some participants saw the experience as vital for their recovery, appreciating the opportunity to stabilize their condition and reduce medication: *“The acute hospitalization process really helped me. It was like a reset. Since I was admitted, I started taking a much lower dose, and it’s been an incredible change”* (P02). However, others recalled traumatic experiences, particularly related to the facilities and staff attitude, linked to dehumanization. These were often described as moments in which stigma was not only felt but embodied through institutional practices: “*They called security, tied me to a stretcher, and locked me in a dark room* and shouted at me and treated me badly*... It was terrifying. The courtyard was narrow and gray, with a high wall, like the ones in prisons*” (P10).

Participants highlighted the critical importance of proper medication monitoring. Some noted that unmonitored dosage reductions could trigger psychotic episodes, emphasizing the need for careful adjustments. Others expressed concern about the danger of certain medications sharing how tragic outcomes occurred when these medications were misused: *“Sometimes, medications are not properly controlled, and that can lead to tragic outcomes, such as using them to commit suicide”* (P07). Some also mentioned that coercive measures, while necessary in certain situations, could be seen as a threat when used improperly (mainly as routine or preventive tools).

*“If you’re a little violent, they use preventive measures to stop you from hurting anyone, including yourself”* (P07).

This theme reveals how healthcare experiences are deeply entangled with stigma—not only in how individuals are treated, but in how they anticipate and interpret these encounters. Issues of continuity, safety, and trust are not merely clinical; they reflect broader social narratives about schizophrenia and the individuals who live with it.

### Surviving mental health struggles

This theme explores the internal and external challenges faced by individuals living with schizophrenia, as well as the strategies they develop to cope and maintain their well-being. On the one hand, participants described how self-stigma, low self-esteem, and emotional distress significantly impact the way individuals view themselves and their condition. Feelings of guilt, shame, and fear often lead to denial of diagnosis and concealment of the illness. On the other hand, resilience emerges as a key theme in overcoming adversity. The support from family, friends, and therapeutic resources, as well as self-acceptance and empowerment, are crucial in helping individuals maintain their well-being and autonomy despite their struggles. This theme emphasizes the strength and coping strategies individuals develop in the face of mental health challenges.

#### Shadows of the Self.

Self-stigma emerged as a central barrier to acceptance and recovery. Many participants internalized negative societal beliefs about mental illness, which significantly affects their self-esteem and emotional well-being.

*“I realized I had stigma, but I didn’t really associate it with stigma. I thought of it as something normal. I even had self-stigma, thinking to myself, ‘What am I going to do now? Am I not going to be able to get ahead? Am I going to end up on the street?’”* (P02).

Others expressed fear of losing control, linking their self-perception to broader public portrayals: *“I know I am a sane person, but sometimes I fear, what if one day I lose my mind?”* (P08).

This self-stigmatization leads some to hold themselves back in various areas of life. *“It’s true that I recognize I often self-stigmatize and that holds me back from attending a social event or gathering, just because I know I have a mental disorder, even though I won’t do anything wrong. But just knowing that I have a disorder, it makes me hold back”* (P04).

Fear of how others perceive their diagnosis is another prevalent concern.

*“Because you think, maybe if I tell them I’m taking medication, I might lose friendships, or maybe I won’t, but you always have that uncertainty of, ‘What will happen?’ Not just socially, but also in work or educational settings”* (P02).

The fear of being judged also leads many to conceal their diagnosis.

*“I can’t tell a neighbor, ‘I have a mental disorder, I have schizophrenia.’ Because of the public image, it’s seen as dangerous, aggressive... I don’t know, that’s why I don’t tell just anyone”* (P04).

*“They’ve been giving a very negative image to those of us who actually have mental health issues, and that’s it, we need support. But with television, people with prejudices just reinforce them even more and start saying, ‘Well, I’m clear about it, mental problem, violent, murderer,’ and maybe we have friends and sometimes they ask about the association, and I say, ‘It’s an association for doing crafts,’ I don’t explain anything, no, I refuse. Because they automatically treat you differently”* (P10).

Some individuals even go as far as describing their condition in less stigmatized terms to redefine their condition in socially acceptable terms, such as anxiety or depression, to avoid the negative implications associated with schizophrenia. *“I never talked about schizophrenia. I always said I had anxiety and depression”* (P05).

#### Resilience in the face of adversity: embracing self-acceptance and support.

Despite the weight of stigma and internal struggle, participants also shared stories of resilience, built through personal growth and the support of others. These experiences reflect the dynamic and non-linear nature of recovery, in which both internal and external resources play a key role.

Peer associations and support networks emerged as crucial resources in navigating life with schizophrenia. Many described these spaces as non-judgmental environments where they could connect with others facing similar challenges, break isolation, and strengthen their sense of identity.

*“The help from the association I joined has been essential in my recovery and in understanding the experiences of others”* (P01).

*“The GAM (Mutual Aid Group) is a big help in destigmatizing mental health, and it also helps me express myself and socialize”* (P03).

These experiences fostered a sense of belonging, encouraging participants to reframe their diagnosis as just one aspect of who they are, rather than a defining feature.

In parallel, self-acceptance was highlighted as a turning point in the recovery process.

By coming to terms with their condition, individuals often find personal growth and empowerment. Accepting their diagnosis allows them to view it as part of themselves, rather than something that limits them.

*“I’ve become stronger by working on positive aspects, and learning to live with my condition, understanding it’s part of who I am”* (P01).

This mindset helps them lead fulfilling lives, maintain jobs, engage in hobbies like sports, and build strong relationships, and find enjoyment in daily routines demonstrating that self-acceptance is a key factor in overcoming adversity.

*“I lead a normal life now, I work, I engage in sports, I have my circle of friends, and I enjoy my routine”* (P01).

Some participants also acknowledged the importance the role of non-human support systems, such as pets, in maintaining structure and emotional balance: *“I have pets, and they help me cope; they give me company and help me maintain my daily routines”* (P07).

Through these networks and practices, many individuals have learned to overcome adversity, embracing a new sense of autonomy and empowerment in their journey with schizophrenia. These testimonies reflect not only the possibility of recovery but also the importance of human connection, self-awareness, and safe spaces in transforming adversity into personal growth.

### Shifting the narrative: Understanding and reversing the stigma of mental illness

This theme addresses the need for a shift in how mental health issues are perceived and portrayed. The media often sensationalizes mental illness. In contrast, efforts to rebuild the public image of mental health emphasize the importance of accurate, empathetic portrayals. Increasing media presence, raising awareness about available resources, and encouraging the use of influencers can help promote a more informed and positive understanding. Additionally, improving communication skills in professionals and fostering greater societal awareness will contribute to dismantling the stigma surrounding mental illness and promoting a more supportive environment. This theme underscores the importance of changing the narrative and fostering a society where mental health is understood with empathy and respect.

#### Rebuilding the public image of mental health.

When participants were asked what proposals, they would offer to modify the public image of mental health -especially schizophrenia- several key ideas emerged. One of the primary suggestions was to increase media presence and utilize impactful platforms to raise awareness.

Participants recognized the importance of using national media, not just local press or television, to disseminate information about mental health in a sustained and accessible manner.

*“Here, when something like World Mental Health Day or similar events happen, it’s important to go beyond just local media”* (P10).

Participants believe that this broader approach could help normalize mental health discussions, reduce misinformation, and reach a wider audience.

Raising awareness about available resources was another critical point. Many participants felt that the public lacks knowledge about the resources available for mental health support.

*“There are many resources, but people often don’t know about them. If they did, they might be more willing to seek help”* (P06).

Making these resources more visible and accessible was seen as a way to counteract stigma and reduce the fear associated with asking for support.

Influencers were also seen as an important tool to improve the public perception of mental health. Public figures who openly discuss their mental health struggles can make a significant impact.

*“For example, a famous journalist recently spoke openly about his mental health struggles, and I think that had a big impact”* (P08).

Such openness by public figures can help normalize mental health issues and encourage others to seek support without fear of judgment.

Finally, real-life interactions were also highlighted as essential for improving the public image. Participants emphasized that personal experiences could shift perceptions and break down stereotypes.

*“People who really know you won’t judge you, but when they don’t know you, they may not understand. Real contact can help change that”* (P07).

Personal stories allow others to see the humanity behind mental health conditions, helping to dispel myths.

#### Transforming communication: advocating for empathy and accuracy in mental health.

Participants also highlighted the importance of ensuring that communication professionals, such as journalists and media personnel, are better equipped to engage with mental health topics. They emphasized that these professionals play a critical role in shaping public discourse and either reinforcing or dismantling stigma.

Improving the communication skills of these professionals was a key point. One participant noted also the need for better training for police officers, who often interact with individuals during psychotic episodes in public spaces, some of which are even reported on television.

*“I think the police need more specific training, especially since they interact with people in such delicate moments when they’re experiencing a psychotic episode”* (P01).

Additionally, some participants shared their experience with media interviews, noting how the interviewer expressed fascination and treated them as if they were a spectacle, asking intrusive questions with morbid curiosity.

*“In interviews, I’ve been treated like some kind of monster, with the interviewer fascinated and asking, ‘What happened to you? What was your delusion like?’ It felt like they were looking for drama”* (P01).

These situations were experienced as deeply uncomfortable and dehumanizing, highlighting the urgent need for respectful, informed, and non-sensationalist approaches to mental health reporting.

All these testimonies underscore the need for better media training to prevent sensationalizing mental health issues and to create more respectful and accurate portrayals. Participants felt that a shift in how stories are selected and told could contribute to long-term cultural change. Training in this area can help reduce stigmatizing language and actions, fostering more positive interactions.

Promoting mental health literacy was a recurrent theme throughout the discussions. Many participants agreed that better public education on mental health could go a long way in dismantling stigma.

*“There should be more public awareness campaigns at the national level to educate people about mental health and its complexities”* (P03).

A better-informed public would be less likely to hold harmful stereotypes and more likely to support those living with mental health conditions.

Finally, participants advocated for a more positive portrayal of schizophrenia, one that emphasizes the resilience and recovery of individuals living with the condition. *“There needs to be more positive representation in the media, showing that people with schizophrenia can lead fulfilling lives”* (P06).

This type of representation would not only reduce the stigma but also inspire others to seek help and manage their conditions effectively.

## Discussion

This study aimed to explore the impact of the public image of mental disorders on the health-illness process and the daily life of individuals diagnosed with schizophrenia. The results confirm that media representations play a fundamental role in shaping stigmatizing attitudes towards individuals with schizophrenia, a finding consistent with previous research that highlights how media contribute to reinforcing negative stereotypes and perpetuating prejudices [14,16]. The portrayal of people with schizophrenia as violent or incapable of leading a normal life directly influences how society perceives these individuals [25], which in turn negatively affects their self-esteem and the social opportunities available to them [25,26].

One of the most significant findings was the experience of self-stigma among participants, where individuals internalize the negative societal attitudes toward their illness. This internalization of public portrays was found to contribute not only to greater difficulty in seeking professional help and lower adherence to treatment [9] but also to a pervasive sense of being judged and rejected by society. This self-stigma, in turn, exacerbates feelings of isolation and alienation, impacting the individuals’ overall mental health [27,28]. However, a crucial aspect of this study, which distinguishes it from others, is its focus on the social stigma that individuals with schizophrenia face outside of the clinical setting. The results highlight how societal views and media portrayals not only shape individuals’ self-concept but also directly influence their willingness to seek help. The stigma surrounding schizophrenia, particularly the association with violence or incapacity to lead a normal life, as frequently depicted in films, creates an environment in which people are reluctant to engage with health services, often delaying treatment or avoiding it altogether. These perceptions affect individuals from pre-diagnosis through treatment, underscoring the importance of addressing stigma not just after diagnosis, but throughout the entire health-illness process. Such societal stigma, including discrimination and fear of being judged, can be as damaging as the illness itself, impacting individuals’ overall health trajectory. These experiences, as reported by participants, also reflect dynamics described in classical sociological theories of stigma.

In particular, our findings can be interpreted through the lens of Goffman’s labeling theory [29], which posits that societal reactions to certain conditions or identities can produce secondary deviance. Several participants expressed that receiving a diagnosis placed them in a socially discredited category, and that from that moment on, they were treated differently—not only by strangers, but also by friends and professionals. They noted the emotional and symbolic weight of receiving a diagnosis. These experiences contribute to what has been termed the ‘hidden burden’ of mental illness stigma [10], where everyday interactions become colored by assumptions about danger, incapacity, or unpredictability. In fact, some participants accounts likened the experience of being diagnosed with schizophrenia to being seen as a criminal or a failure, especially when facing obstacles in areas such as housing access. These added difficulties reinforce this idea—indicating that individuals with schizophrenia may face a social penalty akin to that experienced by those with criminal records, simply for being labeled with a psychiatric diagnosis.

This illustrates how stigma is not only interpersonal but also institutional—embedded in the policies, practices, and systems that govern everyday life. This aligns with what Yang et al. [4] refer to as structural stigma, where institutional arrangements themselves reinforce the marginalization of people with mental disorders.

The study also identified social support as a crucial factor in managing the illness, not only from a medical standpoint but also in relation to the stigma associated with schizophrenia. Family and friends provide a protective role by offering emotional support and acting as advocates in social interactions where stigma may arise. This aligns with research that asserts the importance of support networks in fostering resilience among people living with severe mental disorders [30,31]. However, participants highlighted that the stigma surrounding their condition often complicates their relationships, with some family members and friends struggling to fully accept the diagnosis or fearing social judgment. Thus, the public image of schizophrenia, shaped by media portrayals, extends beyond the individual, affecting their family and friends. These loved ones may also internalize negative societal perceptions, leading to misunderstandings and difficulties in providing support, further isolating the individual and complicating their social interactions. While the lack of institutional support and the precariousness of access to health services were also significant issues raised, the emotional and social backing from close relationships was seen as essential to navigating the stigma they faced.

Professional interaction was another area participants identified as a key factor in their experiences, The public image of schizophrenia not only influences how society perceives individuals with this diagnosis but may also shape the attitudes of professionals who are part of that same society. They indicated that some professionals who interact with individuals diagnosed with schizophrenia, including healthcare providers and law enforcement personnel, should be targeted for anti-stigma interventions to promote a more supportive and understanding approach [3]. Indeed, they noted that the stigma held by healthcare professionals can affect the therapeutic relationship and the quality of care provided. There is evidence suggesting how stigmatization within care settings can negatively impact treatment outcomes, a finding mirrored in our participants’ accounts of distrust and dissatisfaction with health services [6]. Specifically, many participants felt that the stigma associated with schizophrenia impacted their experiences during diagnosis, treatment, and specially hospitalization. This therapeutic relationship is essential in mitigating the effects of stigma on individuals with mental disorders, with empathy and respect from healthcare professionals helping to reduce the negative impact of stigma on treatment adherence and self-esteem [32].

This suggests that professionals, despite being trained to understand mental health, are not immune to dominant narratives, and their perceptions may be shaped by broader cultural messages, including media portrayals and stigmatizing peer discourse. This professional bias further illustrates how stigma is embedded across multiple layers of society, from the public sphere to institutional and clinical settings. There are other possible factors contributing to promote negative conducts in clinical settings, as previous studies have suggested that exposure to stigmatizing peer attitudes in clinical environments can contribute to a perpetuation of bias, particularly in early-career professionals [6,13]. For that reason, participants emphasized the need for healthcare professionals to receive training that goes beyond clinical skills, focusing specifically on empathy and cultural competence. This would enable professionals to engage with mental health conditions in a more holistic way, avoiding the reinforcement of negative stereotypes that can intensify the stigma patients face.

The lack of continuity in care, largely due to frequent staff rotations and insufficient follow-up, was a major concern expressed by participants. They felt that this instability not only hindered their treatment but also deepened the emotional impact of the stigma they encountered. Participants pointed out how films often portray psychiatric treatment in a negative light, emphasizing harsh and dehumanizing practices, such as involuntary hospitalization and the use of force by healthcare professionals. These skewed images shape the public’s perception of both the disorder and the treatment process, creating an environment where patients are hesitant to trust healthcare providers. This disruption increased feelings of distrust and alienation, particularly when interacting with new healthcare providers. The constant change in professionals contributed to difficulties in building rapport and maintaining consistent care, which made it harder for participants to feel understood and supported in their treatment journey. This dynamic creates a barrier to trust, reinforcing the cycle of alienation and reluctance to seek ongoing care.

Another important point raised by participants was the gender difference in the perception of stigma. Women diagnosed with schizophrenia appear to face a double stigma due to both their condition and gender. Previous research emphasizes the double vulnerability faced by women with schizophrenia, who experience stigma not only for their mental illness but also for societal expectations associated with gender roles [33]. In this case, it was mainly male participants who emphasized the double stigma experienced by women. It is difficult to find an explanation as this aspect is still poorly explored and understood. Previous studies pointed that individuals suffering from a mental illness may expect greater stigma when the symptoms align with gender-based stereotypes, when they cannot meet gendered norms and, that men normally show greater discriminatory behaviors towards individuals with psychiatric disorders [34]. The relation between stigma and gender should be further addressed in future research to better understand the dynamics of gender in the stigma associated with schizophrenia in the context of both media portrayals and professional interactions with patients.

Some participants also reflected on how wealth or celebrity status can act as a buffer against stigma. Famous individuals who publicly disclose their diagnosis were perceived as being treated with more leniency. This observation aligns with the *parasocial contact hypothesis*” [35], which suggest that when a celebrity reveals they are experiencing a mental health condition, fans --who feel a one-sided emotional connection with them (parasocial bond)—tend to show reduced their stigmatizing attitudes. They interpret the revelation differently than if it came from an unknown person, even if both individuals suffer from the same condition [36].

But the most novel and relevant aspect of this study lies in the changes suggested by participants to improve the public image of schizophrenia. These suggestions go beyond media representation to encompass aspects of communication within the health system and societal support. Participants emphasized the need for a more positive and balanced portrayal of schizophrenia in the media, one that includes stories of recovery and normalcy. This shift is crucial, as the portrayal of individuals with schizophrenia leading fulfilling lives could help reduce stigma and improve the willingness of other individuals to seek help [37,38]. In this sense, participants suggested that raising awareness about mental health resources could be enhanced by the involvement of influencers. By sharing personal experiences and promoting a more accurate and empathetic image of mental health, influencers have the potential to challenge stereotypes and reduce stigma, especially among younger audiences [37]. Specifically, the literature highlights how celebrity disclosures can positively influence public attitudes, help-seeking behaviors, and mental health awareness, particularly when the celebrity is perceived as credible, relatable, and authentic [39,40].

As previously mentioned, impact of these disclosures often depends on the audience’s identification with the celebrity and the perceived similarity between them. Parasocial relationships—one-sided emotional bonds formed through media exposure—can increase receptivity to such messages, helping reduce stigma by humanizing the condition [36]. However, the effectiveness of celebrity disclosures is not universal. Some individuals may perceive celebrities as too distant from their own lived experiences, which may inadvertently reinforce the idea that “ordinary” people with mental health conditions remain marginalized. This phenomenon, known as subtyping, has been widely documented in social psychology literature [41,42]. It suggests that when individuals are exposed to atypical members of a stigmatized group—such as highly successful celebrities—their stereotypes may remain unchanged and instead leave broader prejudices unaddressed, as these cases are seen as exceptions rather than representative of the group as a whole.

Therefore, while celebrity status can help challenge public stigma—especially when disclosure is perceived as genuine and supported by personal narratives—its impact may vary depending on the audience’s ability to identify with the individual. To enhance the reach and effectiveness of anti-stigma efforts, it is essential to combine these initiatives with broader structural change and to amplify authentic stories from a range of individuals with lived experience [43].

In this regard, recent literature supports the inclusion of both traditional celebrities and microcelebrities—social media influencers or public figures with smaller yet highly engaged followings—as they may connect more directly with diverse audiences and foster a sense of relatability that traditional celebrities sometimes lack [44].

In addition to media representation, participants proposed raising awareness about the available mental health resources. They pointed out that many people do not know where to turn for help, which often results in delayed or avoided treatment. By increasing public knowledge about available resources and services, individuals would feel more supported by society, making it easier for them to engage in their health-illness process from diagnosis onwards. However, providing information alone is not enough. Factors such as mental health knowledge, stigma, and trust in professionals also play a key role in whether individuals seek help. Therefore, raising awareness should be part of a broader approach to address these barriers [45].

Although, the study was conducted within a specific cultural context, there is evidence of similar findings in other countries. Stigma surrounding mental health can vary across societies, yet studies like Shahwan et al. [37] have reported comparable results in different settings, suggesting that the proposed changes to the public image of schizophrenia may have broader applicability. However, further research in diverse contexts is still needed to confirm whether these findings hold universally or if adaptations based on cultural differences are necessary.

### Limitations

As a qualitative study, this research provides valuable insights into the experiences and perceptions of individuals with schizophrenia. However, some limitations should be considered when interpreting the findings.

While qualitative studies typically focus on in-depth exploration of a smaller number of cases, the findings may not reflect the experiences of all individuals with schizophrenia. The inclusion of participants from different regions or backgrounds could provide a broader perspective on the issue of stigma and public image. In particular, all participants in this study were residing in an urban area at the time of the interviews. Although the sample was diverse in age, gender, and clinical background, the lack of rural participants may limit the transferability of the findings. Prior research has indicated that stigma toward mental illness can differ by geographic context, with rural communities often reporting greater perceived stigma than urban ones [46,47]. Additionally, future studies could aim to explore the experiences of individuals with other mental health disorders to determine if the findings are specific to schizophrenia or applicable to a wider range of conditions.

Another limitation is the self-reported nature of the data. As participants shared their experiences through interviews, there is a possibility of social desirability bias or reluctance to disclose certain aspects of their experiences. Participants may have downplayed or exaggerated certain feelings or events to conform to perceived social norms. Although care was taken to ensure an open and non-judgmental interview environment, the subjective nature of qualitative data always leaves room for potential bias. To mitigate this, future research could include triangulation with other data sources, such as healthcare providers’ perspectives or observational data, to further enrich the findings.

Lastly, while the focus on the health-illness process provided valuable insights into the experiences of individuals living with schizophrenia, this study did not explore the impact of these findings on healthcare outcomes. Future research could investigate whether changes in public perception and professional attitudes result in improved treatment adherence, service user satisfaction, or mental health outcomes, thus establishing a more direct link between stigma reduction and better health outcomes.

In conclusion, while this study offers a deep understanding of the impact of public image on individuals with schizophrenia, these limitations suggest that further research is needed to confirm and expand upon these findings in different contexts and with a larger and more diverse sample.

## Conclusion

The changes proposed by participants have significant implications for the health-illness process. From the moment prior to diagnosis, the way society and healthcare professionals perceive and treat individuals with schizophrenia affects their entire experience with the illness. The negative portrayal of schizophrenia, both in the media and by professionals, contributes to delays in seeking help, difficulties in accepting the diagnosis, and challenges in adhering to treatment. By shifting public perceptions and improving the way healthcare professionals interact with patients, the stigma that surrounds schizophrenia could be reduced, leading to a more supportive environment for individuals navigating their health-illness journey.

Finally, the findings of this study underline the importance of addressing stigmatizing media representations and the need for better education for both the public and healthcare professionals. These changes are essential to improving the quality of life for individuals with schizophrenia and facilitating their recovery and reintegration into society. The novel contributions of this study, particularly the insights into the potential impact of positive media portrayals and the need for professional training, offer a valuable foundation for future efforts to reduce stigma and enhance mental health care.

### Declaration of generative AI and AI-assisted technologies in the writing process

During the preparation of this work, the authors used ChatGPT to review and improve the English language of the article. After using this tool, the authors reviewed and edited the content as needed and take full responsibility for the content of the publication.
